# Enabling Real-Time 3D Display of Lifelike Fingerspelling in a Web App

**DOI:** 10.1007/978-3-030-58805-2_5

**Published:** 2020-08-12

**Authors:** Jami Montgomery, John McDonald, Eric Gong, Souad Baowidan, Rosalee Wolfe

**Affiliations:** 8 Institute Integriert Studieren, JKU Linz, Linz, Austria; 9 Jack Baskin School of Engineering, UC Santa Cruz, Santa Cruz, CA USA; 10 Dipartimento di Meccanica, Politecnico di Milano, Milan, Italy; 11 Support Centre for Students with Special Needs, Masaryk University Brno, Brno, Czech Republic; 12 Georgetown University, Washington, DC, USA; 13 DePaul University, Chicago, IL USA; 14 King Abdulaziz University, Jeddah, Saudi Arabia

**Keywords:** Sign language, Avatars, Fingerspelling, Educational tools

## Abstract

Fingerspelling receptive skills remain among the most difficult aspects of sign language for hearing people to learn due to the lack of access to practice tools that reproduce the natural motion of human signing. This problem has been exacerbated in recent years by the move from desktop to mobile technologies which has rendered prior software platforms less accessible to general users. This paper explores a web-enabled 3D rendering architecture that enables real-time fingerspelling on a human avatar that can address these issues. In addition it is capable of producing more realistic motion than prior efforts that were video-based and provides greater interactivity and customization that will support further enhancements to self-practice tools for fingerspelling reception.

## Introduction

Sign language is the preferred form of communication for millions of people around the world [2], and for many signers, written language is a second language. These factors contribute to the many challenges that Deaf and hard-of-hearing people face in day to day interactions with the hearing. For the millions of hearing people who interact with native signers, educational software can be an important part of the learning process and can provide valuable practice opportunities for many critical parts of sign language.

A component of many sign languages is fingerspelling, in which the hands convey the letters of an alphabet from a spoken language. The collection of hand configurations used in fingerspelling for a given spoken language is called a manual alphabet. By sequentially forming handshapes, words in the spoken language may be spelled letter by letter. Many sign languages incorporate fingerspelling as a key link to the spoken language, and while fingerspelling usage varies considerably among sign languages [11], most use fingerspelling for conveying

- Proper names of people and places,
- Loan worlds, such as technical terminology,
- Terms for which there is no commonly agreed-upon sign [1].

Fingerspelling is particularly important when signers are communicating with hearing people. For example, signers most often have sign names, but hearing people they interact with will tend not to have a sign name, and so fingerspelling will often be used for both participants.

In addition, fingerspelling can be an integral part of bi-lingual education for Deaf children, who continue to face challenges in early education, particularly with written forms of spoken languages. In the United States, the average reading level of deaf high school graduates has remained near the fourth-grade level for decades, and studies of early deaf education have shown that increased experience with fingerspelling positively influences a deaf child’s reading ability [9].

Because of these factors, fingerspelling is an important component of both Deaf-hearing communication and early deaf education. Unfortunately, it can also be a difficult skill to learn, particularly for hearing people. This is somewhat ironic since it is the one part of signing that is actually borrowed from their spoken language. The problem lies not in the production of fingerspelling, but rather in its recognition or reception, i.e. reading a word being fingerspelled. Fingerspelling receptive skills are a notorious challenge for students in interpreter training programs and are often cited as the first skill learned but the last skill mastered. This is particularly true in ASL when the fingerspelling is rapid [8].

## Limitations of Current Practice Tools

One important factor that exacerbates the challenge of learning fingerspelling reception is the lack of practice opportunities that are available to students in fingerspelling classes. The two most common practice methods are peer practice, which is practicing with other students, and watching videos of fingerspelling. Peer work between students can be ineffective for learning to read fluent fingerspelling as it involves watching non-native fingerspelling that is much slower and also inexpertly produced.

Video practice tools such as DVDs and https://www.handspeak.com/spell/ practice/ are problematic because of their immutability. They are limited to the words that have been recorded, and the fact that the cost of recording and distributing new fingerspelling recordings can be quite high, particularly for media such as DVD’s, though online distribution does mitigate this somewhat. In any case, for video fingerspelling study, a student can end up learning or memorizing the specific productions of the particular words provided in the video. These limited recordings obviously cannot match the variability of productions they will witness in fluent discourse.

Several attempts have been made at computer and web-based fingerspelling recognition practice tools that attempt to display any fingerspelled word. Unfortunately, most of these are image-based tools such as [14], which provide sequentially shown still drawings or photos. As noted by [15], fingerspelling is conveyed, not by the still shape of the hand, but by its motion as it moves through the shapes in the word. Thus, it is the motion of the fingers that we perceive in a signer, not the individual handshapes when recognizing a word, and therefore, practice software that displays sequences of still pictures will not help students to recognize human fingerspelling motion at fluent speeds.

Increasingly, computer generated avatars have offered a compelling solution to this need as they are capable of producing the infinite variability of fingerspelling productions on demand and at variable speeds. However, prior practice tools leveraging 3D avatars have had several issues that hindered wider adoption including

- The display of an isolated 3D hand. When learning to recognize fingerspelling, it is important to have the whole visual field of the person or avatar [5]. In fact most still-frame tools have the same issue as they only show pictures or drawings of isolated hands
- Motion that did not prevent self-intersection of the fingers, such as seen in [3, 12]. Human fingers will naturally move to avoid each other in transitions between letters, and any tool that does not prevent collisions in the fingers will not provide the realistic fingerspelling motion needed by students.
- Motion that was limited only to the arm and fingers of the avatar. The previously presented mobile drill and practice tool presented in [13] made great strides in providing natural motion of the fingers along with motion of the signer’s arm for double letters using a single pre-rendered video of an avatar to display any word. Unfortunately, because of the nature of the video-clip splicing playback technique that was used in the app, the torso of the avatar was not able to move, which does not match the natural motion of a human signer. This resulted in a robotic appearance for the full avatar even as the motion of the hand was fluid and natural.
- Limited platform support. Even in the case of the full body avatar in [13], it was implemented as a native app that had the limitations of running only on Windows devices. Also, due to its technology relying on a large pre-recorded video of individual transitions between letters, it required a substantial amount of device storage. This is potentially problematic on some smaller devices like smartphones.

This paper introduces a novel web-based fingerspelling practice app that builds on the prior Fingerspelling Tutor technology in [13] by addressing these deficiencies. It does this by encapsulating the Fingerspelling Tutor experience in a web-app and implementing a 3D avatar display that provides fully interactive animations of one-handed fingerspelling in real-time to enhance the self-practice experience. Further, the app has been designed to support a variety of sign languages, and to allow the fingerspelling of any word in the chosen sign language. In addition, it features interactive client control of the avatar with full camera movement so that fingerspelling may be viewed at a variety of speeds and viewing angles to simulate different conversation settings.

The previous Fingerspelling Tutor software was built, as described in [6] with the participation of interpreter training students, and its continued development has been driven by feedback from this target user group [16]. The current effort has also been driven by requests from users of the prior software, who indicated that wider platform support, more realism in the avatar’s motion and increased interactivity with the avatar display.

## Building a Better Practice Tool

To achieve a ubiquitously supported client, the new practice tool utilizes WebGL2 [4] to provide a responsive user interface that renders and animates the fingerspeller on the client’s device. This mitigates the need to download or stream video and thereby significantly and reduces mobile bandwidth usage. The design requirements were:

1. Minimal data storage requirements on client device
2. Minimal initial data transmission between server and client
3. Minimal data transfer from client device to server when requesting an fingerspelling
4. Minimal data transfer from server to client when playing an animation

To accomplish all of these requirements, the new web app has been designed so that the server leverages the existing services from the prior Fingerspelling tutor software detailed in [13], which supported fingerspelling in a variety of sign languages in a native Windows app. This prior system utilized smooth motion controllers for the avatar’s joints, and an exhaustive collection of inter-letter poses to avoid collisions between fingers during transitions to provide natural fingerspelling motions. The web app encapsulates this functionality on a native compiled Windows server but leaves the actual realization of these motions of the avatar to the client.

**Fig. 1.**
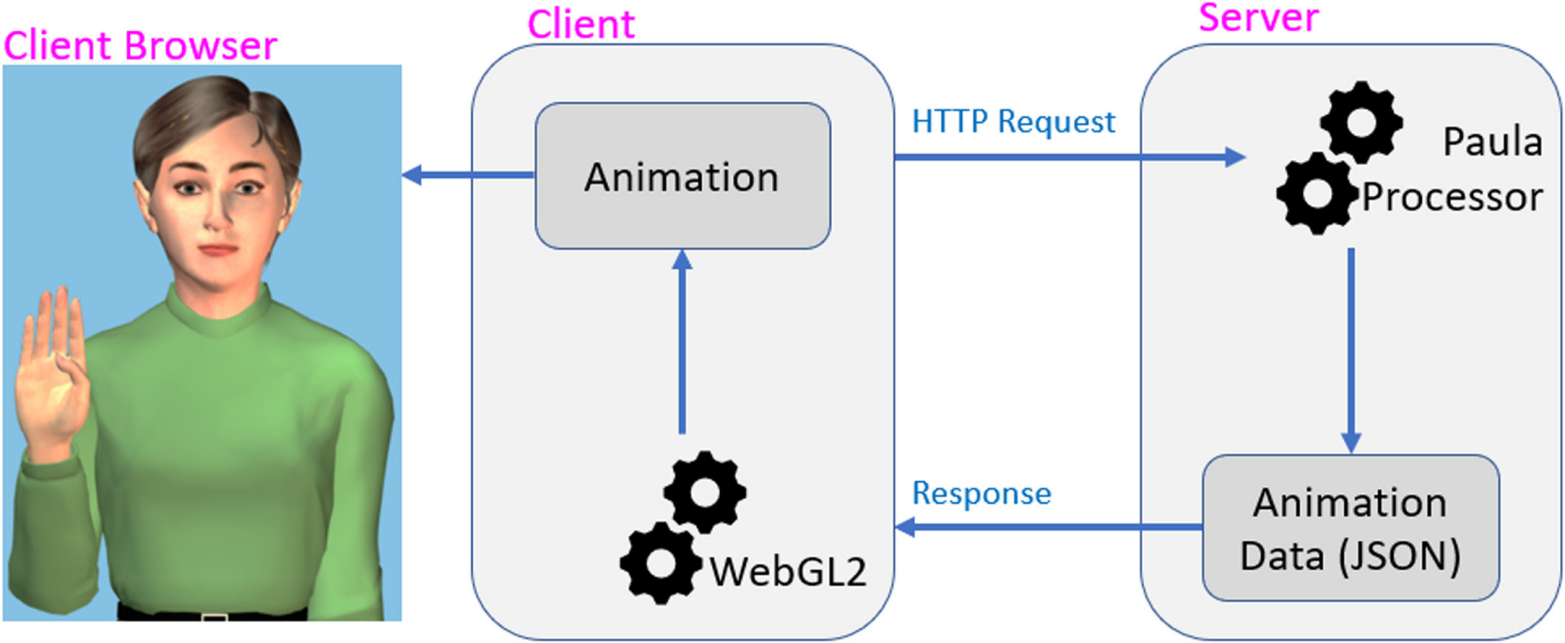
Diagram of client-server architecture

The client provides for interactive rendering and animation of the avatar which is supplied in an FBX format. To support ubiquitous access, the client is written in JavaScript and employs a WebGL2 JavaScript library called three.js for rendering [10], which is provided under the MIT software license. Communication between the client and server uses the standard HTTP2 protocol, and the data transmitted is encoded using the standard JSON format. A diagram of the architecture of this web app is provided in Figure 1.

In this diagram, the client-side application is implemented in JavaScript and relies on a mere eight megabytes to store the avatar data, including all geometry and surface textures. The server is implemented in C# on Windows.

As in our previous fingerspelling tutor, the new app will support a variety of options for input and interaction with the user including allowing the user to ask the avatar to fingerspell a word that the user types in, and also support a quiz facility with words coming randomly from a variety of word lists. In either case, the communication protocol for the system will require the client to request a word for fingerspelling and for the server to receive the word.

In order to minimize complexity in the JavaScript client, the server applies a variety of techniques to compose the animation data for the avatar

1. Sequencing the individual hand shapes in time, including the finger movements necessary for certain letters (e.g.. J and Z in ASL).
2. Computing interpolations that avoid collisions between the fingers and thumb via a library of artist-animated motion paths.
3. Adding motion of the arm for double letters either, e.g.. bounce or slide to the side depending on the sign language
4. Adding continuous ambient motion to the avatar to avoid a frozen robotic appearance

Scheduling these effects on the avatar is complex because many of these processes can affect the same joints and are computed with very different motion controls. For example, sequencing handshapes is done with traditional key-frame animation where settings for all of the hand’s joints are applied at a given time. Then layering onto this ambient motion, which is an application of Perlin noise [7], requires a more complicated avatar structure than we desired on the client side.

In keeping with goals 1–4 above, the server pre-computes the animation on each of the avatar’s joints by using a more complex internal representation and then sends frame-by-frame data to the avatar for playback. Since the client has full data for every bone at each frame, it does not have to perform interpolation and thus very simple animation controllers can be used, improving performance on slower hardware. Of course, by sending frame-by-frame data, we are increasing the amount of data being sent vs only sending keyframes, but since the Avatar has 25 bones in the torso and arm, the data for the body for a 10 s video is still less than 200 kilobytes as a clear-text JSON file, which can be reduced to less than 50k by using a binary encoding. Compare this to approximately 40 MB for a 10 s VGA resolution video at 30 fps. Thus, the web app requires less than 0.5% of the bandwidth required to transmit a video of the same animation, which results in a quick response time, even when internet connectivity is poor.

## Conclusion and Next Steps

The new web app presented here builds upon prior Fingerspelling Tutor technology by providing greater platform access, greater naturalness in fingerspelling motion for receptive skill practice and allowing greater freedom for exploring fingerspelling contexts such as different points of view. The results reported in this paper concern an important technological advancement for a Fingerspelling reception practice tool which was designed and developed with input from the interpreter training community.

Once the new 3D display is completely integrated into the functionality of Fingerspelling Tutor, the software will be further tested with sign language students to determine the extent to which the added interactivity impacts student experience and learning.
